# Molasses wastewater treatment and lipid production at low temperature conditions by a microalgal mutant *Scenedesmus* sp. Z-4

**DOI:** 10.1186/s13068-017-0797-x

**Published:** 2017-05-02

**Authors:** Chao Ma, Hanquan Wen, Defeng Xing, Xuanyuan Pei, Jiani Zhu, Nanqi Ren, Bingfeng Liu

**Affiliations:** State Key Laboratory of Urban Water Resource and Environment, Harbin Institute of Technology, P. O. Box 2614, 73 Huanghe Road, Harbin, 150090 China

**Keywords:** Molasses wastewater, Lipid production, Low temperature, *Scenedesmus* sp. Z-4

## Abstract

**Background:**

Simultaneous wastewater treatment and lipid production by oleaginous microalgae show great potential to alleviate energy shortage and environmental pollution, because they exhibit tremendous advantages over traditional activated sludge. Currently, most research on wastewater treatment by microalgal are carried out at optimized temperature conditions (25–35 °C), but no information about simultaneous wastewater treatment and lipid production by microalgae at low temperatures has been reported. Microalgal growth and metabolism will be inhibited at low temperature conditions, and satisfactory wastewater treatment performance will be not obtained. Therefore, it is critical to domesticate and screen superior microalgal strains with low temperature adaptability, which is of great importance for wastewater treatment and biodiesel production.

**Results:**

In this work, simultaneous wastewater treatment and lipid production were achieved by a microalgal mutant *Scenedesmus* sp. Z-4 at the low temperature conditions (4, 10, and 15 °C). The results showed that algal growth was inhibited at 4, 10, and 15 °C compared to that at the optimal temperature of 25 °C. However, decreased temperature had no significant effect on the total cellular lipid content of algae. Importantly, lipid productivity at 10 °C was compromised by more net energy output relevant to biodiesel production, which demonstrated that the low temperature of 10 °C was favorable to wastewater treatment and energy recovery by *Scenedesmus* sp. Z-4. When molasses wastewater with optimal COD concentration of 8000 mg L^−1^, initial inoculation ratio of 15%, and C/N ratio of 15 was used to cultivate microalgae, the maximum removal rate of COD, TN, and TP at 10 °C reached 87.2, 90.5, and 88.6%, respectively. In addition, lipid content of 28.9% and lipid productivity of 94.4 mg L^−1^ day^−1^ were obtained.

**Conclusions:**

*Scenedesmus* sp. Z-4 had good adaptability to low temperature conditions, and showed great potential to realize simultaneous wastewater treatment and lipid production at low temperatures. The proposed approach in the study was simple compared to other wastewater treatment methods, and this potential novel process was still efficient to remove COD, N, and P at low temperatures. Thus, it had a vital significance for the wastewater treatment in low temperature regions.

**Electronic supplementary material:**

The online version of this article (doi:10.1186/s13068-017-0797-x) contains supplementary material, which is available to authorized users.

## Background

Energy shortage and environmental pollution have been the worldwide issues, which are mainly caused by heavy dependence on fossil fuels [1]. Thus, alternative and sustainable biofuels (such as biodiesel, bioethanol, and biohydrogen) have attracted more and more attention due to their renewable and environmentally friendly properties. Biodiesel derived from microalgae has opened up a novel avenue to the development and utilization of clean bioenergy [2]. As one of the most promising sources for producing biodiesel, microalgae have its incomparable advantages compared to other biodiesel producers [3]. On one hand, it could be cultivated on non-arable lands, thus avoiding the competition with food crops [4]. On the other hand, the fast growth rate and high lipid productivity of microalgal cells are suitable for the biodiesel production. Although there are considerable advantages, the high cost of biodiesel produced by microalgae still limits their application, and it is necessary to be further reduced to meet industrial production requirement [5]. Therefore, in practice, microalgae have been cultured with wastewater instead of synthetic medium [6, 7], to achieve simultaneous nutrient removal and energy recovery from useless and cheap substrates.

Currently, activated sludge (AS) process is widely used in biological treatment of various kinds of wastewater. However, microalgae are also able to grow well in nutrient-rich wastewater, thus making a contribution to the reduction of pollutants and environmental sustainability [8]. Due to its potential to efficiently remove the nutrients and organics under heterotrophic/mixotrophic mode, along with the production of potentially value-added products, wastewater treatment using microalgae is gaining more attention [9]. The organic carbon and pollutants in the wastewater could be converted into cellular components such as carbohydrates and lipids, via the metabolism of microalgal cells. In addition, microalgae can assimilate inorganic nitrogen (N) and phosphorous (P) for cell growth, which is well approved as an efficient and economical method for wastewater treatment and has been widely used in bioremediation process [10, 11].

In traditional treatment process, microorganisms existing in wastewater treatment systems (activated sludge, biofilm, etc.) belong to mesophilic bacteria, and their optimal growth temperature is 20–37 °C. However, in low temperature regions, especially in the northern regions of China, the wastewater temperature is usually below 10 °C in the winter or spring, and the average annual temperature in Harbin City is just 4.4 °C [12]. In such conditions, the cell growth and metabolism activity of these mesophilic microorganisms are inhibited, which has a negative effect on the degradation ability of microorganisms, thus efficiency of wastewater treatment decreases in large extent. To solve this problem, increasing sludge return and hydraulic retention time (HRT), reducing sludge loading, and even artificial heating are adopted in engineering [13]. These greatly increase project investment and operation cost, but satisfactory performance of wastewater treatment is not obtained. Therefore, research on improving wastewater treatment efficiency at low temperatures will be of important theoretical and realistic significance. Microalgae with good environmental adaptability and metabolic plasticity are promising to be applied in wastewater treatment at low temperatures, compared to activated sludge process. However, the information about wastewater treatment by microalgae at low temperatures has not been reported.

Temperature is one of the most important environmental factors determining microalgal growth, which strongly affects the biomass, lipid productivity, and lipid composition of microalgae, and the effects of temperature are species dependent [14, 15]. So far, raceway ponds and tubular photobioreactors have been widely used to culture microalgae in a large scale [16, 17]. However, in these systems, temperature has to be maintained between 20 and 30 °C in order to obtain ideal microalgal growth and lipid accumulation because lower temperatures result in lower biomass. Most of the researches on the effect of temperature on algae are performed at controlled and optimized conditions [18, 19], or typical stress conditions which could be beneficial to the accumulation of some metabolites [20]. Therefore, it is important to estimate and understand the cell growth and lipid accumulation at low temperatures when subjected to low sub-optimal temperatures such as 4–15 °C, and to domesticate and screen excellent microalgal strains with low temperature adaptability for wastewater treatment and biodiesel production.

In our previous research, anaerobic sludge and microalgae were co-cultured to enhance the nutrient removal and energy conversion from starch wastewater [21], and the highest COD, TN, and TP removal rate all reached above 80% when simulated starch wastewater was used as the substrate. This study will provide a novel potential for wastewater treatment by microalgae. As one of the most seriously polluted wastewater in the food industry, molasses wastewater with high concentration of COD and complex components could cause serious environmental pollution on the ecosystem [22]. Molasses wastewater by conventional anaerobic microbial treatment needs to maintain the temperature of the reactors above 30 °C [23], which increases the cost greatly because of massive energy input. In addition, complexity and high COD concentration limit the treatment and energy production. Molasses wastewater contains plenty of nutritional components such as polysaccharides, nitrate, and phosphate [24], which were essential for microalgal growth. Therefore, in this work, microalgal cells were cultivated at low temperatures for the treatment of molasses wastewater, aiming to achieve simultaneous energy recovery and nutrient removal and provide a new potential approach for wastewater treatment at low temperatures.

*Scenedesmus* sp. Z-4 was an oleaginous microalgal mutant, which was screened in previous experiment [25] and it has good temperature adaptability after temperature gradient domestication. The object of this study is to investigate the cell growth and lipid accumulation of *Scenedesmus* sp. Z-4 at low temperature (4–15 °C) and determine the optimal low temperature for molasses wastewater treatment by the analysis of energy input and output related to the process of biodiesel production. The effects of culture conditions including molasses wastewater concentration, inoculation ratio, and C/N ratio on lipid accumulation and nutrient removal were also investigated.

## Methods

### Microalgal species and culture conditions

The microalgal mutant *Scenedesmus* sp. Z-4 was used in all the following experiments. This mutant was cultured in Erlenmeyer flasks of 250 mL containing BG-11 medium of 150 mL (10% microalgal inoculum, V/V) under white fluorescent light (3000 lux with a light/dark cycles of 12 h/12 h) in batch culture, which was supplemented with 10 g L^−1^ glucose and adjusted to pH of 7.0 and autoclaved at 121 °C for 15 min. To ensure the metabolic activity at low temperatures, microalgal cells were cultured by decreased temperature gradient domestication of 25, 20, 15, 10, and 4 °C, respectively. The temperatures in this experiment were set as 4, 10, and 15 °C, respectively, and 25 °C of the optimal temperature was served as the control. In the experiments of molasses wastewater treatment, the glucose in the medium was replaced by waste molasses, to provide the carbon source and nutrients for microalgal growth and lipid accumulation.

### Molasses wastewater

Waste molasses was obtained from a sugar refinery factory located in Harbin, China. The initial COD concentration, total nitrogen (TN), and total phosphorus (TP) of waste molasses were approximately 514,000, 458, and 67 mg L^−1^, respectively. The main components of waste molasses are listed in Additional file 1: Table S1. To avoid the negative effect of excess COD concentration on microalgal growth, the molasses wastewater was diluted to achieve a final COD concentration of about 5000 mg L^−1^. To supply sufficient nitrogen and phosphorus sources for cell growth, TN and TP should be maintained at 600 mg L^−1^ [26] and 75 mg L^−1^ (equal to the content in BG-11 medium) by additional NaNO_3_ and K_2_HPO_4_ into the medium. In order to determine the optimal molasses concentration, original waste molasses was diluted to 5000, 8000, 10,000, and 15,000 mg L^−1^, respectively. Because the ratio of COD/TN in initial waste molasses has exceeded 1000, NaNO_3_ had to be added into the medium to obtain final C/N ratios of 5, 10, 15, and 20, respectively. Fresh microalgal inoculum volumes of 7.5, 15, 22.5, and 30 mL were transferred into molasses wastewater of 150 mL, to make sure the inoculation ratios were 5, 10, 15, and 20%, respectively.

### The calculation of energy input and output

The energy input consisted of (i) energy for increasing water temperature, (ii) energy for microalgal cultivation and harvesting, and (iii) energy for biodiesel production. The energy output mainly came from (iv) biodiesel combustion. The calculation was based on 1 L medium.

(i) The energy input for increasing water temperature (EI_1_) was calculated as the following equation.
  1 $${\text{EI}}_{1} = c_{w} \times m \times \left( {t - t_{0} } \right),$$
  where *c*_w_ was the specific heat capacity of water, equal to 4.2 × 10^3^ J kg^−1^ °C^−1^. *m* referred to the mass of 1 L medium, which was assumed as 1 kg. *t* was the temperature applied in this study, which were 5, 10, 15, and 25 °C, respectively. Considering that the temperature of winter and spring in North China was usually below 0 °C, *t*_0_ was set as 0 °C to simplify this calculation.
(ii) The energy input for microalgal cultivation and harvesting (EI_2_) is shown in Eq. (2).
  2 $${\text{EI}}_{ 2} {\text{ = EH}} \times {\text{biomass}} \times {\text{lipid content}} \times 9 0 {\text{\% }}$$
  Here, an assumption was made that 90% of total fatty acids could be converted into biodiesel from the produced lipids, according to a previous study [27]. EH was the energy required for microalgal cultivation and harvesting per ton of biodiesel preparation, equal to 2288 kWh ton^−1^ (1 kWh = 3.6 × 10^6^ J) [28]. Biomass and lipid content depended on the determination results at different temperature conditions.
(iii) The energy input for biodiesel production (EI_3_) was obtained using the equation below.
  3 $${\text{EI}}_{ 3} {\text{ = EC}} \times {\text{biomass}} \times {\text{lipid content}} \times 9 0 {\text{\% }},$$
  where EC referred to the energy consumption per ton of biodiesel production, which was equal to 9 kWh ton^−1^ according to a previous report [29]. The transesterification efficiency was also 90% as stated above.
(iv) The energy output (EO) in the process of biodiesel combustion was calculated as the Eq. (4).
  4 $${\text{EO = }}Q \times {\text{biomass}} \times {\text{lipid content}} \times 9 0 {\text{\% }}$$
  Here, *Q* was the energy release per kg of biodiesel combustion, equal to 39.51 MJ/kg [30].

### Analytical methods

Biomass was collected by centrifugation, followed by washing of normal saline, and then weighted on an electronic scale after drying at 105 °C until constant weight was obtained. Lipid was extracted and determined as per the procedures in the literature [31]. Briefly, the lipids were extracted from dried biomass with a solvent mixture of chloroform/methanol (2:1, v/v). The supernatant was recovered and the same process was carried out two times for the complete extraction of lipids. After the determination of biomass and lipid content, lipid productivity could be calculated according to the following equation.

5 $${\text{Lipid productivity }}\left( {{\text{mg L}}^{ - 1} {\text{day}}^{ - 1} } \right){\text{ = biomass}} \times {\text{lipid content/}}t$$

Here, *t* was the cultivation time (*d*), and equal to 7 days in our experiments. This was due to the maximum lipid productivity obtained on the 7th day (Additional file 1: Figure S1).

The fatty acid composition analysis was performed using the methods previously described [32]. The solvent phase was combined, dried, and weighed. The chemical oxygen demand (COD), total nitrogen (TN), and total phosphorus (TP) were measured according to the methods described previously [33–35]. The glucose concentration in the culture broth was determined by the method in our previous study [26]. Polysaccharide content was measured by the phenol and sulfuric acid method described in a previous literature [36]. Protein content was obtained using UV–vis based on the Bradford method [37]. All experiments were conducted in triplicate, and data were recorded as the mean with standard deviation (SD). Fluorescence microscope (BX51/TF, Olympus Co., Japan) was applied to observe cells stained with Nile Red (0.1 mg mL^−1^ acetone solution), and the excitation and emission wavelength were 530 and 575 nm, respectively. The pictures of microalgal cells and oil bodies were acquired randomly from at least 10 cells per sample, and typical images are shown here.

## Results and discussion

### The cell growth and lipid production of *Scenedesmus* sp. Z-4 at low temperature conditions

**Fig. 1:**
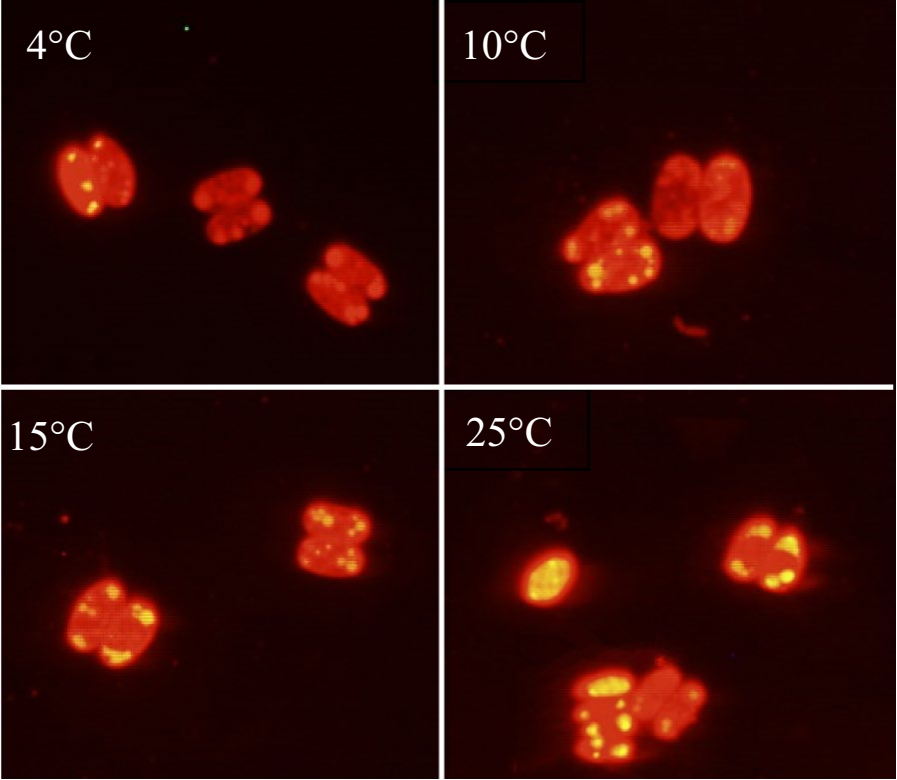
Fluorescence micrograph of microalgal strain Z-4 staining with Nile red at different temperatures

The microalgae strain *Scenedesmus* sp. Z-4 with Nile red staining at different temperatures of 4, 10, 15, and 25 °C are shown in Fig. 1a–d. Microscopic observation showed that fluorescence intensity and the amount of lipid droplets in microalgal cells increased with the increase of culture temperature from 4–25 °C. Low fluorescence intensity was observed at 4 °C, indicating that low content of lipid existed in the cells of strain Z-4 (Fig. 1a). This result was not surprising because 4 °C was usually used as the temperature for culture preservation and microorganisms almost ceased to grow at this temperature.

**Fig. 2:**
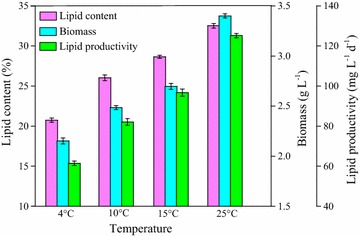
Biomass and lipid accumulation of strain Z-4 at different temperatures

To further investigate quantitatively the cell growth and lipid production at low temperatures, the biomass, and lipid productivity were determined at 4, 10, 15, and 25 °C, respectively. As shown in Fig. 2, with the temperature increasing from 4 to 25 °C, lipid content, biomass, and lipid productivity all gradually increased. The obtained maximum lipid productivity of 125 mg L^−1^day^−1^ at the optimal temperature of 25 °C was evidently higher than that at low temperatures of 4, 10, and 15 °C (63, 78, and 86 mg L^−1^ day^−1^, respectively). This was due to the inhibition of cellular metabolism at low temperatures, especially low enzymatic activities responsible for cell growth and lipid accumulation [38].

Furthermore, the low temperature had a more significant effect on biomass compared to the lipid content. There was a great difference in the final biomass at the optimal temperature (3.4 g L^−1^ at 25 °C), compared to that at low temperatures of 4, 10, and 15 °C (1.8, 2.6, and 2.8 g L^−1^, respectively). However, the lipid contents at the low temperatures were 20, 26, and 28% for 4, 10, and 15 °C, respectively, which was still comparable to that at stress temperature in some literatures [39, 40]. This result showed that the lipid content may be an inherent characteristic for the oleaginous mutant *Scenedesmus* sp. Z-4, which was not easily affected by the environmental factors such as the temperature. Even though the biomass and lipid productivity at the temperatures of 10 and 15 °C were lower than that at 25 °C, they were considerable enough for the potential of wastewater treatment.

Because not all the fatty acids were suitable for transesterification to biodiesel, the compositions of fatty acids at 4, 10, and 15 °C were examined to determine whether the fatty acids produced at low temperatures were suitable for the biodiesel production. The main fatty acids at different temperatures were from C14 to C20, which indicated the dominance of long-chain fatty acids (Table 1).

**Table 1:** The compositions of fatty acids (mass percentage) of microalgal strain Z-4 at different temperatures

Compositions of fatty acids	4 °C	10 °C	15 °C	25 °C
C 15:0 2OH	0.67 ± 0.12	–	0.47 ± 0.13	0.33 ± 0.11
C 15:0 3OH	0.20 ± 0.07	–	0.22 ± 0.07	–
C 16:0	35.98 ± 2.03	38.51 ± 3.28	38.98 ± 3.56	39.51 ± 3.71
C 17:1 w8c	–	0.22 ± 0.10	–	0.19 ± 0.07
C 18:1 w7c	0.43 ± 0.09	0.25 ± 0.09	0.43 ± 0.11	–
C 18:1 w9c	25.92 ± 1.64	27.92 ± 2.34	28.43 ± 2.62	29.18 ± 2.53
18:2 w6,9c	18.22 ± 1.35	13.50 ± 1.13	14.24 ± 1.64	15.50 ± 1.42
18:3 w6c (6,9,12)	3.86 ± 0.59	1.90 ± 0.31	1.86 ± 0.28	1.58 ± 0.34
C 20:0	0.30 ± 0.11	0.28 ± 0.07	0.29 ± 0.10	0.26 ± 0.08
C 20:1 w9c	0.17 ± 0.04	0.18 ± 0.06	0.17 ± 0.08	0.20 ± 0.09
Saturated fatty acids	47.41 ± 2.33	52.55 ± 3.35	53.41 ± 3.86	55.56 ± 3.90
Unsaturated fatty acids	53.59 ± 3.71	47.44 ± 4.03	46.59 ± 4.73	44.44 ± 4.45

The compositions of main fatty acids at different temperatures were almost the same, especially the four fatty acids, including palmitic acid (C 16:0), stearic acid (C 18:0), oleic acid (C 18:1 w9c), and linoleic acid (C 18:2 w6,9c), accounted for more than 90% of total fatty acids. The tiny distinctions lay in more palmitic acid (C 16:0) and oleic acid (C 18:1 w9c) and less linoleic acid (C 18:2 w6,9c) at the temperatures 10, 15, and 25 °C, compared to that at 4 °C. It should be noted that more linolenic acid (C 18:3 w6c) was obtained at the temperature of 4 °C. It has been reported that more saturated fatty acids (C 16:0 and C 18:0) and less unsaturated fatty acids (C 18:2 and C 18:3) were suitable for the biodiesel production [41]. This result implied that the compositions of fatty acids at the temperature of 4 °C may not be applicable in transesterification.

**Fig. 3:**
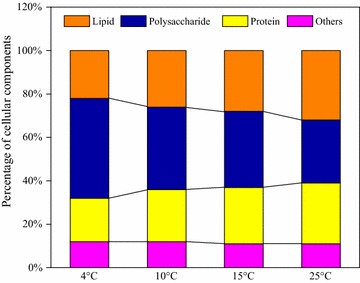
Percentage of main cellular components of microalgal strain Z-4 at different temperatures

To further investigate the variation of cellular components at different temperatures, the contents of polysaccharide, lipid, and protein at the temperatures of 4, 10, 15, and 25 °C were determined (Fig. 3). Result showed highest percentage of polysaccharide accounted for 46% at the temperature of 4 °C, which was in agreement with a previous study that starch accumulation was observed under growth-inhibiting conditions, to protect cellular structures and increase the microalgae survival probability [42]. Furthermore, with the temperature increasing from 4 to 25 °C, the content of lipid and protein gradually increased, and the content of polysaccharide decreased. It was not in consistent with a previous study by Ho et al. [43], which showed that carbohydrate and lipid both accumulated in case of nitrogen starvation. This could be attributed to severe nitrogen deficiency caused by the prolonging of cultivation time, therefore, carbohydrate and lipid were largely produced. However, with the temperature increasing from 4 to 25 °C in this study, the negative influence of stress environment on microalgal cells started to weaken. Cell growth and metabolic activities enhanced with the increase of temperature and more energy substances (such as polysaccharide) could be utilized to the accumulation of lipid.

**Fig. 4:**
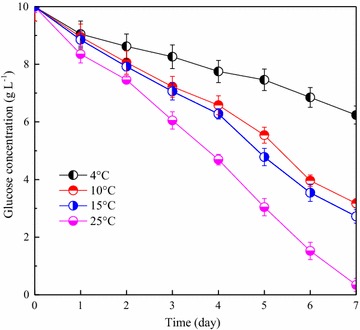
Time course profiles of glucose concentration for strain Z-4 at different temperatures

As well known, the microorganisms at low temperatures could not utilize the substrate well, which was due to lower cellular activity. The glucose consumption at different temperatures in the course of microalgal growth is clearly presented in Fig. 4. In the cultivation process of 8 days, utilization rate of glucose was only approximately 40%, which suggested that inefficient substrate uptake was unfavorable to cell growth and lipid accumulation. In contrast, glucose concentration was almost zero on the 7th day at the temperature of 25 °C. Approximately 80% of glucose was consumed at the low temperatures of 10 and 15 °C, which was still an acceptable result.

Here, a summary was made to compare the biomass, lipid productivity, compositions of fatty acids, cellular components, and glucose consumption at the temperatures of 4, 10, 15, and 25 °C. As shown in Additional file 1: Table S2, a conclusion could be drawn that 4 °C was not suitable for wastewater treatment because the cell growth, lipid accumulation, and substrate utilization at this temperature were not satisfactory.

### The energy conversion efficiency and feasibility of wastewater treatment by *Scenedesmus* sp. Z-4 at low temperature conditions

#### Energy conversion efficiency at different temperature

As stated above, the desirable lipid productivity, fatty acids composition, and intracellular components were obtained at the low temperatures of 10 and 15 °C, even though they were not as high as that at the optimal temperature. However, maintaining the suitable temperature of 25–30 °C for the microalgal growth meant more energy input. If net energy output did not significantly increase at the optimal temperature, it would not be recommended in terms of economics. It should be noted that the wastewater temperature was lower than 10 °C in the winter and spring of northeastern regions in China, and the growth and metabolism of most mesophilic functional microorganisms were largely inhibited at the low temperatures. Although the artificial heating could improve the efficiency of wastewater treatment, the energy consumption and operation cost would greatly increase.

A heat value comparison of energy input and output in 1 L pure BG-11 medium was calculated at different temperatures. As shown in Table 2, the energy input was mainly from three parts, increasing water temperature, microalgal cultivation and harvesting, and biodiesel production. The first part dominated the total energy input. The results showed that with the temperature increasing from 4 to 25 °C, more energy input was required to increase the temperature of cultivation system, however, the differences in energy demand for microalgal cultivation and harvesting and biodiesel production at different temperatures were almost negligible, leading to a great difference in total energy input at various temperatures (19.92, 47.22, 69.26, and 112.38 kJ, respectively). On the other hand, considerable energy output derived from biodiesel was not obtained with the increasing of temperature, and 33.74 kJ of energy output at 25 °C was not obviously higher than that (22.43 kJ) at 10 °C. The overall effect was that net energy output and the ratio of output/input significantly decreased, from 4 to 25 °C. This demonstrated that more energy input was needed in higher temperature, which was not cost effective from the perspective of economics.

**Table 2:** The calculation of energy input and output (kJ) for microalgae biodiesel process at different temperatures

Temperature (°C)	Energy input^a^				Energy Output	Net energy output	Output/input
	Water heating EI_1_	Cultivation and harvesting EI_2_	Biodiesel preparation EI_3_	Sum of energy input	Biodiesel		
4	16.8	3.11	0.012	19.92	13.42	−6.50	0.67
10	42	5.20	0.020	47.22	22.43	−24.79	0.48
15	63	6.23	0.025	69.26	26.87	−42.39	0.39
25	105	7.35	0.031	112.38	33.74	−78.64	0.30

^a^The data were calculated based on 1L pure medium

Moreover, when wastewater was used to cultivate microalgae, lipid productivity would decrease to some extent, which had a negative effect on biodiesel yield. However, a large amount of energy was still needed to maintain the cultivation temperature condition, leading to a further decrease of the ratio between energy output to input, which was not favorable to wastewater treatment and energy recovery. If satisfactory wastewater treatment performance was obtained at the low temperature, the treatment method should be proposed. Highest ratio of output/input was obtained at 4 °C, but the biomass and lipid accumulation were too low, which have been discussed above. Beside 4 °C, best ratio of output/input occurred at 10 °C. By comprehensive consideration of lipid productivity, the cost of wastewater treatment and the ratio of energy output/input, 10 °C was the best low temperature condition.

#### Wastewater treatment by *Scenedesmus* sp. Z-4 at low temperature conditions

**Fig. 5:**
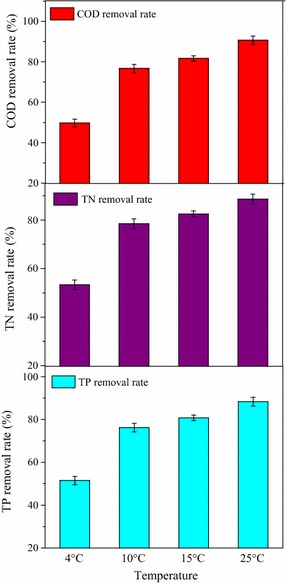
Wastewater treatment performance of COD, TN, and TP removal rate by *Scenedesmus* sp. Z-4 at different temperatures

Furthermore, this work tested the COD, TN, and TP removal by *Scenedesmus* sp. Z-4 at the temperatures of 4, 10, 15, and 25 °C (Fig. 5). After several domestication of molasses wastewater, the highest removal rate of COD, TN, and TP was obtained at the optimal temperatures of 25 °C, 86.6, 84.5, and 83.3%, respectively. For the performance at 4 °C, it showed poor removal rates with COD, TN, and TP at the temperature, which may be due to the inactive cellular metabolism at this temperature. Higher removal rates of COD, TN, and TP were still obtained at the low temperatures of 10 and 15 °C, even though slightly lower than that at 25 °C. These results showed satisfactory wastewater treatment efficiency at the low temperature of 10 °C, which showed the feasibility of simultaneous energy recovery and nutrient removal from molasses wastewater.

In the following work, to enhance the wastewater treatment performance and simultaneous lipid production by strain Z-4, the optimization of initial molasses concentration, inoculation ratio, and ratio of C/N in the medium were carried out, and their effects on lipid production and removal rate of COD, TN, and TP were also investigated.

### Treatment performance of molasses wastewater at the low temperature of 10 °C

#### The effect of initial molasses concentration on lipid production and wastewater treatment

The concentration of organic matters in wastewater treatment system had a significant effect on the microbial growth and metabolism [43]. Excessively high or low concentration was unfavorable to microbial growth and accumulation of metabolites.

**Fig. 6:**
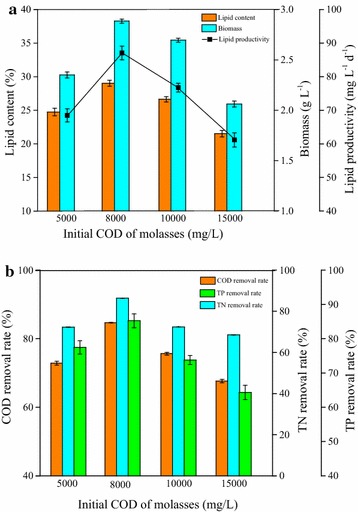
Effect of initial molasses concentration on lipid accumulation and wastewater treatment performance at 10 °C by strain Z-4 **a** Cellular growth and lipid production. **b** COD, TN, and TP removal rate

To investigate the effect of initial molasses concentration on wastewater treatment and lipid accumulation of strain Z-4 at 10 °C, the initial COD of molasses was set as 5000, 8000, 10,000, and 15,000 mg L^−1^, respectively. As shown in Fig. 6a, when the molasses concentration increased from 5000 to 8000 mg L^−1^, biomass/lipid productivity significantly increased from 2.3 g L^−1^/68.2 mg L^−1^ day^−1^ to 2.8 g L^−1^/86.4 mg L^−1^ day^−1^. This suggested that the increase in molasses concentration was beneficial to cell growth and lipid accumulation, which could be attributed to the vigorous metabolism of strain Z-4 at low molasses concentration. However, while the molasses concentration further increased to 10,000 and 15,000 mg L^−1^, biomass and lipid productivity decreased to 2.5 g L^−1^/80.5 mg L^−1^ day^−1^ and 2.1 g L^−1^/72.8 mg L^−1^ day^−1^, respectively. Results indicated that excessive high molasses concentration in the medium had a negative effect on the energy production for strain Z-4.

Furthermore, removal rate of COD, TN, and TP at 10 °C, supplemented with initial molasses concentration of 5000, 8000, 10,000 and 15,000 mg L^−1^ was studied (Fig. 6b). When the concentration increased from 5000 to 8000 mg L^−1^, removal rate of COD, TN, and TP evidently increased from 73.2, 72.4, 77.5 to 85.6, 84.4, 83.7%, respectively. The further increase of molasses concentration would result in the gradual decrease to 68.6, 69.5, and 64.5% at 15,000 mg L^−1^, which showed a similar tendency to cellular growth. It should be noted that more lipid or COD removal rate could be obtained compared with other reported studies [44, 45] when molasses wastewater was used as the substrate, which verified the potential of strain Z-4 for simultaneous lipid production and wastewater treatment.

In this study, an appropriate molasses concentration could favor the wastewater treatment and energy production by strain Z-4, and thus molasses with 8000 mg L^−1^ of COD would be adopted in the following experiments.

#### The effect of inoculation ratio on lipid production and wastewater treatment

The inoculation ratio (V/V) had a remarkable influence on the lag phase of microorganisms [46]. When the inoculation ratio was high, microbial community could enter into logarithmic phase quickly. However, excessive initial inoculation ratio would be the limiting factor for microbial growth, which had an important effect on the metabolism of microorganism, including lipid accumulation. Therefore, it was crucial to investigate the correlation between microalgal inoculation ratio and wastewater treatment performance.

**Fig. 7:**
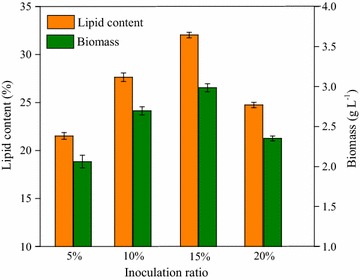
Effect of inoculant volume on microalgal cell growth and lipid production of strain Z-4 at 10 °C

To determine the optimal inoculation ratio, initial inoculation ratio of 5, 10, 15, and 20% were chosen for the experiments. Biomass and lipid productivity obviously increased from 2.2 g L^−1^/61.4 mg L^−1^ day^−1^ to 2.9 g L^−1^/92.3 mg L^−1^ day^−1^ when inoculation ratio increased from 5 to 15% (Fig. 7). This could be attributed to the sufficient nutrients in the culture system, which was beneficial to the microalgal growth and lipid production, when the inoculation ratio was less than 15%. However, while inoculation ratio further increased to 20%, biomass and lipid productivity visibly decreased to 2.3 g L^−1^ and 65.5 mg L^−1^ day^−1^, respectively. The reason could be deficient nutritional components which inhibited microalgal growth and lipid accumulation.

**Fig. 8:**
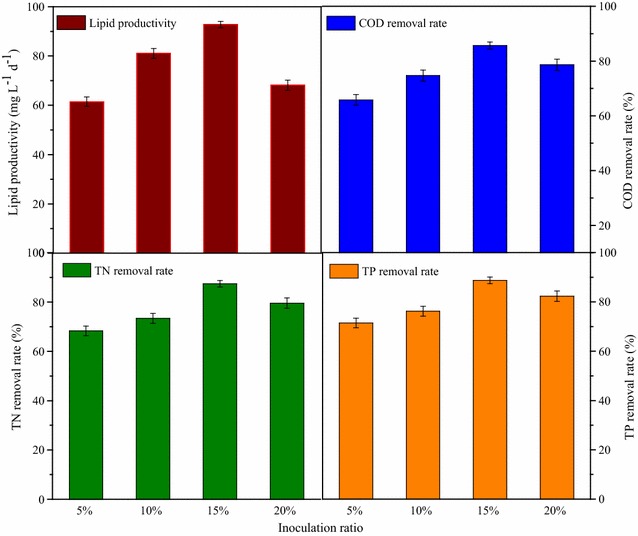
Effect of inoculant volume on wastewater treatment performance of COD, TN, and TP removal rate by strain Z-4 at 10 °C

Wastewater treatment performance of COD, TN, and TP removal rate was in accordance with cell growth and lipid accumulation (Fig. 8). The optimal removal rate of COD, TN, TP was 86.4, 87.9, and 85.2%, respectively, which was obtained under the initial inoculation ratio of 15%. In previous studies, microalgae were mixed with anaerobic sludge [21] or activated sludge [47] to treat various wastewaters and the optimal mixed ratio was investigated. However, in this study, it was demonstrated that strain Z-4 only could reach better wastewater treatment performance under the optimized inoculation ratio.

It should be noted that the optimal microalgal inoculation ratio was 15% for molasses wastewater system, which was higher than that for BG-11 medium (10%). This may be due to the relatively complex components of molasses, compared to the pure medium, and thus demanded more microalgal cells to activate the cultivation system.

#### The effect of ratio of C/N on lipid production and wastewater treatment

**Fig. 9:**
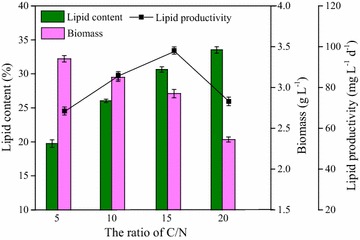
Effect of C/N ratio on cellular growth and lipid production of strain Z-4 at 10 °C

The ratio of C/N was of great importance to cellular metabolism, which regulated the balance between microalgal growth and lipid accumulation. As shown in Fig. 9, when the ratio of C/N was 5, the highest biomass of 3.4 g L^−1^ was obtained, while the lipid content and productivity were relatively low. The highest lipid content was determined at the ratio of C/N of 20, but the biomass was lowest. This result was similar with previous studies [48], which demonstrated that low C/N was beneficial to cell growth and biomass accumulation, while high ratio of C/N was in favor of lipid synthesis. Therefore, in a two-stage cultivation of microalgae, low C/N should be applied in the first stage to harvest more biomass, and high C/N was followed in the second stage to enhance the lipid accumulation, which could be an effective approach to obtain more biomass and lipid in two-stage system by adjusting C/N. However, when the ratio of C/N was at 15, highest lipid productivity of 94.4 mg L^−1^ day^−1^ was obtained even though biomass of 3.0 g L^−1^ and lipid content of 28.9% were not the optimal, which should be attributed to the balance between cell growth and lipid accumulation caused by the appropriate ratio of C/N.

**Fig. 10:**
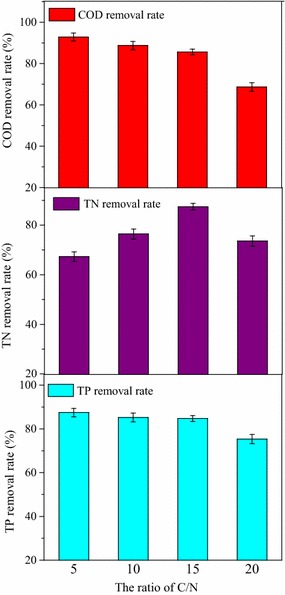
Effect of C/N ratio on wastewater treatment performance by strain Z-4 at 10 °C

The wastewater treatment performance was also investigated under different ratios of C/N. As was indicated in Fig. 10, when C/N was at low ratios of 5 and 10, higher COD removal rate was observed, which was demonstrated by more biomass accumulation; however, low TN removal rates of 67.5 and 74.3% were caused by excessive nitrogen concentration. When C/N was high at the ratio of 20, insufficient nitrogen was the main limiting factor, which inhibited cell growth and wastewater treatment performance. Thus, it could be concluded that the optimal ratio of C/N was 15, by combining the results of microalgal metabolism with the removal rate of COD (87.2%), nitrogen (90.5%), and phosphorus (88.6%).

Here, a short conclusion could be reached that the maximum removal rate of COD, TN, and TP at 10 °C were 87.2, 90.5, and 88.6%, respectively, when molasses wastewater with optimal COD concentration of 8000 mg L^−1^, initial inoculation ratio of 15%, and C/N ratio of 15 were applied in the experiments. Furthermore, the lipid content of 28.9% and lipid productivity of 94.4 mg L^−1^ day^−1^ were also obtained.

### Comparisons between the proposed approach in this study with other wastewater treatment processes

#### Comparison with current molasses wastewater treatment techniques

As shown in Table 3, various molasses wastewater treatment techniques were compared. Traditionally, molasses wastewater was treated by activated sludge (AS), and microalgae and ozonation have been coupled to enhance treatment performance [45, 49]. On one hand, aeration was essential to maintain the metabolic activity of aerobic bacteria in AS process, which needed large amount of energy input. On the other hand, the combined wastewater systems were not easy to control, and COD removal rates were 78 and 66.5%, which were not satisfactory results. Even though higher removal rate of COD (98.2%), TN (90.2%), and TP (85.5%) were obtained in another combined system, which could be mainly attributed to the treatment of AFBR (anaerobic fixed bed reactor), and microalgae pond was only a post-treatment unit. Undoubtedly, extra treatment units would increase the cost of wastewater treatment. Microbial fuel cells (MFC) were also applied to simultaneous molasses wastewater treatment and electricity generation [50]. However, biodiesel derived from microalgae was a better alternative, compared to electricity recovery. Importantly, these treatment methods were conducted at about 30 °C, to ensure microbial metabolism and activity. Once the temperature decreased to a low level, the performance of wastewater treatment would significantly decrease. However, in this study strain Z-4 was cultivated with molasses wastewater at the low temperature of 10 °C, and lipid production and wastewater treatment efficiency achieved the desired results.

**Table 3:** Comparison of molasses wastewater treatment by different methods (Trophic mode of microalgae in table is heterotrophic cultivation)

Methods	Temperature	Removal performance	References
Microalgae and activated sludge	25 ± 1 °C	COD ~78%, TN ~35%	[45]
Ozonation and activated sludge	25 °C	COD ~66.5%	[50]
Microbial fuel cells (MFC)	Not given	COD ~79.8%	[51]
Single chamber MFC	Not given	COD ~89–90%	[52]
AFBR and microalgae pond	27–32 °C	COD ~98.2%, TN ~85.5%	[53]
ABR	Not given	COD ~90%	[54]
BioH_2_–BioCH_4_–MFC integrated system	35 °C	COD ~98%	[55]
UASB	35 °C	COD ~91.2%	[56]
Microalgae alone	10 °C	COD ~87.2%, TN ~90.5%, TP ~88.6%	This study

#### Comparison with traditional wastewater treatment processes

In traditional wastewater treatment processes, the simultaneous removal of COD, nitrogen, and phosphorus was difficult to realize in a single stage of conventional wastewater treatment. In order to further remove nitrogen and phosphorus from the wastewater, the new process such as anaerobic/anoxic/oxic (AAO) process had to be added in traditional activated sludge process [57]. However, the control of combined systems was usually complicated, and additional treatment units had to be added, which were mentioned above. In this study, better COD, TN, and TP removal were achieved in a single microalgae unit, which had significant advantage over activated sludge process.

Sludge separation and disposal were also troublesome in traditional wastewater treatment. On one hand, the sludge had to be separated with water in activated sludge process, which was often time- and labor-consuming [58]. However, the microalgal cultures could be easily obtained by simple setting or centrifugation to recycling and reutilization because of interaction and self-aggregation of microalgal cells. On the other hand, the disposal of massive sludge formed in wastewater treatment process was complicated. In this process, large amount of greenhouse gases (N_2_O, CH_4_, and CO_2_) were also produced following the conversion of organic matters in wastewater [59]. However, wastewater treatment by microalgae could avoid the problems above, because no sludge took part in this treatment unit and the produced CO_2_ could be utilized by microalgal cells.

In a word, the simultaneous lipid production and nutrient removal from molasses wastewater at low temperature conditions was of great significance, providing a novel approach for wastewater treatment in winter or spring of some region or country, such as in the north region of China. In the future work, microalgal cultivation will be combined with the treatment of other wastewater at low temperature, to verify the feasibility of microalgae wastewater treatment system.

## Conclusions

The growth and lipid accumulation of microalgae mutant *Scenedesmus* sp. Z-4 were inhibited at the low temperatures of 4, 10, and 15 °C. Although the lower lipid productivity were obtained at 10 °C, less energy input were needed with more net energy output, compared to the optimal temperature of 25 °C. When molasses wastewater was used as the substrate, the COD, TN, and TP removal rate at the low temperature of 10 °C reached 87.2, 90.5, and 88.6%, under optimal condition of COD of 8000 mg L^−1^, inoculation ratio of 15%, and C/N of 15. These results showed that microalgae had a great potential for the simultaneous wastewater treatment and energy recovery in low temperature regions.

## Additional file

**Additional file 1: Figure S1.** Time course profiles of biomass and lipid accumulation for strain Z-4 at 25 °C. **Table S1.** The main components (mass percentage) of waste molasses in this study. **Table S2.** A comparison of cell growth, lipid accumulation, compositions of fatty acids, cellular components and glucose consumption at different temperatures.

## Abbreviations

COD: chemical oxygen demand
TN: total nitrogen
TP: total phosphorus
AS: activated sludge
HRT: hydraulic retention time
AFBR: anaerobic fixed bed reactor
ABR: anaerobic baffled reactor
AAO: anaerobic/anoxic/oxic
MFC: microbial fuel cells
UASB: upflow anaerobic sludge blanket
