# Supplementary material for: Molasses wastewater treatment and lipid production at low temperature conditions by a microalgal mutant Scenedesmus sp. Z-4
Source: Biotechnol Biofuels. 2017 May 2;10:111. doi: 10.1186/s13068-017-0797-x (PMC5414357; doi:10.1186/s13068-017-0797-x)
Supplement: Supplementary file 1 — Additional file 1: Figure S1. Time course profiles of biomass and lipid accumulation for strain Z-4 at 25 °C. Table S1. The main components (mass percentage) of waste molasses in this study. Table S2. A comparison of cell growth, lipid accumulation, compositions of fatty acids, cellular components and glucose consumption at different temperatures. [file 13068_2017_797_MOESM1_ESM.doc]

**Supporting Information**

**Molasses wastewater treatment and lipid production at low temperature conditions by a microalgal mutant *Scenedesmus* sp. Z-4**

Chao Ma, Hanquan Wen, Defeng Xing, Xuanyuan Pei, Jiani Zhu, Nanqi Ren, Bingfeng Liu^*^

*State Key Laboratory of Urban Water Resource and Environment, Harbin Institute of Technology, P.O. Box 2614, 73 Huanghe Road, Harbin 150090, China*

*Email:lbf@hit.edu.cn*

**Fig. S1 Time course profiles of biomass and lipid accumulation for strain Z-4 at 25 °C**

**Table S1 The main components (mass percentage) of waste molasses in this study**

| Components | Percentage |
| --- | --- |
| Dry matter | 78-85 % |
| Total sugar | 48-56 % |
| TOC | 29-36 % |
| TKN | 0.3-2.7 % |
| P_2_O_5_ | 0.03-0.06 % |
| CaO | 0.15-0.75 % |
| MgO | 0.02-0.08 % |
| K_2_O | 2.2-4.2 % |
| SiO_2_ | 0.1-0.5 % |
| AlO_2_ | 0.04-0.06 % |
| Fe_2_O_3_ | 0.005-0.02 % |
| Ash | 4-8 % |
| COD | 514000 mg L^-1^ |
| TN | 458 mg L^-1^ |
| TP | 67 mg L^-1^ |

**Table S2 A comparison of cell growth, lipid accumulation, compositions of fatty acids, cellular components and glucose consumption under different temperatures**

|  | 4 °C | 10 °C | 15 °C | 25 °C |
| --- | --- | --- | --- | --- |
| Biomass (g L^-1^) | 2.1 | 2.5 | 2.7 | 3.5 |
| Lipid productivity (mg L^-1^ d^-1^) | 61 | 78 | 86 | 105 |
| Saturated fatty acids (%) | 46.4 | 52.6 | 53.4 | 55.6 |
| Unsaturated fatty acids (%) | 53.6 | 47.4 | 46.6 | 44.4 |
| Lipid content (%) | 21 | 26 | 28 | 32 |
| Polysaccharide content (%) | 46 | 38 | 35 | 29 |
| Protein content (%) | 20 | 24 | 26 | 28 |
| Glucose consumption (%) | 39 | 78 | 84 | 98 |
